# The influence of a destination’s red cultural atmospherics on tourists’ confidence in Chinese culture

**DOI:** 10.3389/fpsyg.2022.992125

**Published:** 2022-08-16

**Authors:** Xiaoli Zhou, Yulin Guo, Xiaofeng Xie, Chunjuan Liu, Fengying Zhang

**Affiliations:** ^1^Department of History and Tourism Management, Changzhi University, Changzhi, China; ^2^Taihang Institute of Ecological Culture, Changzhi, China; ^3^School of Political Science and Public Administration, Shanxi University, Taiyuan, China; ^4^West China School of Nursing, Nursing Key Laboratory of Sichuan Province, Innovation Center of Nursing Research, West China Hospital, Sichuan University, Chengdu, China

**Keywords:** red tourism, red cultural atmospherics, perception of red education, red cultural identity, Chinese cultural self-confidence

## Abstract

This study explores the development of tourists’ confidence in Chinese culture through red tourism. Partial least squares structural equation modeling (PLS-SEM) was adopted to analyze the survey data collected from red tourists in Wuxiang county, Changzhi, Shanxi province, China to examine the influence of a destination’s red cultural atmospherics on tourists’ confidence in Chinese culture. The results indicated that Wuxiang’s red cultural atmospherics composed of physical atmospherics, spiritual atmospherics and institutional atmospherics significantly influences tourists’ confidence in Chinese culture. Tourists’ perception of red education and their red cultural identity mediate the impact of the destination’s red cultural atmospherics on tourists’ confidence in Chinese culture. These findings contribute to the red tourism literature by providing empirical support for building tourists’ confidence in Chinese culture through red tourism. The empirical results also offer managerial implications for red tourism destinations’ planning and management.

## Introduction

The report delivered by China’s president Xi Jinping at the 19th National Congress of the Communist Party of China clearly pointed out that without a high degree of cultural self-confidence and cultural prosperity, there will be no great rejuvenation of the Chinese nation. In other words, people’s Chinese cultural self-confidence is significantly related to the great rejuvenation of the Chinese nation. Cultural self-confidence is based on the subject’s strong cultural identity (Negy et al., 2003; Dabamona et al., 2021). Chinese cultural self-confidence is essentially Chinese people’ identification with as well as promotion and inheritance of the Chinese culture (Chen, 2016; Yan and Ye, 2022). According to the Outline of the 14th Five-Year Plan (2021–2025) for Tourism Development issued by China’s State Council, China will continue to promote the integration of culture and tourism, to deeply tap into and explain the cultural connotation, to integrate historical culture and modern civilization into the development of tourism, and to turn tourism into a process for tourists to understand Chinese culture and build their Chinese cultural self-confidence (Zhou, 2022).

The Chinese excellent traditional culture, advanced socialist culture and red revolutionary culture are the three main components of Chinese culture and are historical source of Chinese cultural self-confidence (Chen, 2016; Zhang, 2020). Developing red tourism is an important way to innovate and inherit the red revolutionary culture in China (Wall and Zhao, 2017; Tang et al., 2021). By participating in red tourism, tourists can perceive, learn, experience, understand and inherit the national spirit and history displayed by the red revolutionary relics and communist heritage sites (Zhao and Timothy, 2015), which will help tourists build a red cultural identity. On one hand, red revolutionary culture is one of the three main components of Chinese culture. Managers and scholars, therefore, believe that tourists’ red cultural identity will further strengthen their confidence in Chinese culture (Jiang et al., 2021). As a result, Chinese governments at all levels are enthusiastic about prioritizing red tourism products in tourism planning, which helps increase the popularity of red tourism across the country. On the other hand, some red tourism destinations exhibit a wrong tendency in the planning and development where red tourism is developed as a product of “mass tourism” with little consideration of its red cultural connotation, resulting in the serious abuse of red cultural resources in these destinations. As a result, the cultural inheritance and patriotic education of red tourism are barely effective, which is not conducive to the improvement of tourists’ confidence in Chinese culture. As a cultural movement to promote the “red spirit,” red tourism embodies cultural and propagandistic elements (Tang et al., 2021). The red cultural atmospherics in a destination is an important source of its core attraction and competitiveness (Xu and Zhu, 2016). Existing studies have shown that guests’ perception of the cultural atmospherics of different sites is an important factor affecting their attitudes and behaviors (Turley and Milliman, 2000; Heung and Gu, 2012; Dong and Siu, 2013; Lee et al., 2022). In other words, red cultural atmospherics may act as an antecedent to improve tourists’ confidence in Chinese culture. Therefore, this paper aims to explore the development of tourists’ confidence in Chinese culture through red tourism. According to the Stimulus-Organism-Response (S-O-R) model of behavioral psychology, the stimuli from red cultural atmospherics will affect tourists’ psychological processes and trigger the response of Chinese cultural self-confidence in them. As an effective patriotic education strategy, red tourism in China is heavily used as a political initiative to increase tourists’ identification with communism and red revolutionary culture. In other words, red tourism provides tourists with an intense revolutionary atmospherics. When tourists immerse themselves in this red cultural context, they naturally access their patriotic education, which can be quite different from encountering mass tourism products for entertainment purposes and help tourists develop their confidence in Chinese culture. Also, tourists will enrich their knowledge of China’s revolutionary culture and deepen their understanding in Chinese red culture through red tourism, which also helps tourists to improve their Chinese cultural self-confidence. As a result, we explores the development of tourists’ confidence in Chinese culture through red cultural atmospherics. And tourists’ perception of red education and red cultural identity are explored as mediators between red cultural atmospherics and tourists’ Chinese cultural self-confidence.

This paper is expected to contribute to the red tourism literature by providing empirical support for the development of tourists’ confidence in Chinese culture in red tourism destinations. Besides, the empirical results are expected to offer managerial implications for red tourism destinations’ planning and scientific improvement of the red cultural atmospherics. The reminder of this paper is structured as follows. First, an integrative conceptual framework is constructed to address how red cultural atmospherics in a red tourism destination influences tourists’ confidence in Chinese culture and what the psychological mechanism is. Then the methods used are described, and the empirical findings reported. Finally, a general discussion of the findings is provided, theoretical and managerial implications highlighted, and limitations & suggestions for future research pointed out.

## Literature review and research hypotheses

### Literature review

#### Red tourism and Chinese cultural self-confidence

“Red tourism” is a type of tourism related to the Chinese Revolution (Zuo et al., 2016). China’s central government has implemented a series of nationwide strategic such as the National Red Tourism Development Planning (2004–2010; 2011–2015; 2016–2020) to facilitate red tourism development across the country because of its political functions. As a type of culture-themed tourism to inherit revolutionary genes while displaying national spirit and socialist achievements, red tourism is expected to strengthen tourists’ party identification and national identity and to strength tourists’ support for the Chinese government (Zuo, 2014; Zhao et al., 2016; Zhao and Timothy, 2017). Therefore, red tourism is heavily leveraged as a tool for patriotic education in China (Xu, 2015; Tang et al., 2021).

In China, red tourism is considered as a political initiative to increase tourists’ identification with communism and red revolutionary culture. Red tourism scenic spots are places presenting the defining characteristics of China’s nationhood and displaying revolutionary evidence of its independence and prosperity. Tourist attractions in revolutionary sites may play a key role in the formation and maintenance of national identity. Existing literature has qualitatively and quantitatively analyzed the effectiveness of red tourism development in strengthening tourists’ national identity and party identification (Caraba, 2011; Zhao et al., 2016; Zuo et al., 2017). According to Jiang et al. (2021), red tourism is helpful to improve tourists’ red cultural identity and provides a scientific value for the final aim of the red cultural foster. However, few empirical studies tested the path/mechanism by which red tourism increase tourists’ Chinese cultural self-confidence. In addition, a detailed exploration into how red tourism affects tourists’ Chinese culture self-confidence remains lacking. Therefore, it is necessary to empirically explore tourists’ confidence in Chinese culture as a consequence of red tourism. Specifically, this study will focus on exploring the impact of red cultural atmospherics of a destination on tourists’ confidence in Chinese culture.

#### Red cultural atmospherics

The term atmospherics was coined by Kotler (1973) as a marketing tool. He defined atmospherics as the conscious designing of the buying environments to create specific emotional impacts on shoppers to enhance their intention to buy. Therefore, atmospherics is not of equal importance to different sellers. For example, atmospherics is a more relevant marketing tool in service settings than in setting with tangible goods (Uhrich and Benkenstein, 2012). Atmospherics can be captured through consumers’ sensory channels. As a result, ambient conditions, spatial layout, functionality, signs, symbols, and artifacts can all be acknowledged as measurements for atmospherics (Bitner, 1992).

Cultural atmospherics emphasizes the cultural elements in the atmospherics (Xu and Zhu, 2016). In the tourism context, professionals often state that what they sell is “experience” (Heung and Gu, 2012). Particularly, in a destination, what is sold can be seen as “sensory experience.” The core products of a destination include not only the attractions but also the service, the interaction between tourists and local residents, as well as the atmospherics in the destination. Given the integration of culture and tourism, more and more researchers focused on the cultural atmospherics of a destination, and their findings supported the statement that the cultural atmospherics of a destination plays an important role in improving the quality of tourists’ experience in the destination. For example, some researchers showed that perceptions of the cultural atmospherics in a heritage destination are conducive to tourists’ genuine experiences (MacCannell, 1973). And the cultural atmospherics associated with a theme park’s location can also influence tourists’ experiences in the theme park (Wu et al., 2018). Since there are different types of destinations, the characteristics of the cultural atmospherics are different. For example, in Tibet, China, the most distinctive cultural atmospherics is its religious element. Existing research results showed that the sacred religious-cultural atmospherics there can offer tourists a unique and authentic experience (Cui et al., 2014; Zhang and Zhang, 2017). Similarly, the most distinctive cultural atmospherics of red tourism destinations in China is their red cultural atmospherics.

According to Xu and Zhu (2016), red cultural atmospherics is composed of three dimensions: physical atmospherics, spiritual atmospherics and institutional atmospherics. Physical atmospherics, as the foundation of the cultural atmospherics of a tourist destination, refers to the cultural style presented by physical symbols such as attractions, buildings, natural landscapes, snacks and souvenirs. For example, the walls of many villages in Wuxiang County are painted with murals of the revolutionary era, creating strong red cultural atmospherics. Spiritual atmospherics, as the core of the cultural atmospherics of a tourist destination, refers to the spirit and world view displayed by the main stakeholders of a tourist destination. In Wuxiang County, residents have a strong sense of pride in Taihang spirit reflected in their words and deeds. Tourists can perceive the red cultural atmospherics during their interactions with local residents. Institutional atmospherics, as the guarantee of the cultural atmospherics of a tourist destination, refers to various management systems and behavior norms of the stakeholders in the tourist destination.

### Hypotheses development

#### Red cultural atmospherics and tourists’ Chinese cultural self-confidence

In environmental psychology, many scholars constructed theoretical models of individuals’ experiences based on factors associated with their perceptions of environmental atmospherics (Heung and Gu, 2012). As stated before, cultural atmospherics is one aspect of the whole environmental atmospherics. Red cultural atmospherics, manifested in presented revolutionary relics and objects as well as the red tourism destination’s historical background, reflect the destination’s unique local cultural environment and the cultural appeal of red tourism attractions. Scholars believe that although cultural atmospherics is an abstract concept, tourists can nevertheless perceive it with their five senses (Kotler, 1973; Bitner, 1992) and form their personal perception of the cultural environment within red tourism sites. Tourists’ perception of red cultural atmospherics is pivotal to their red tourism experience. Existing studies conceptualized red cultural atmospherics into two dimensions, namely static and dynamic cultural atmospherics. Besides, literature revealed that tourists’ static and dynamic red cultural atmospherics can positively influence their red tourism experience (Tang et al., 2021). Therefore, this paper argues that compared with the entertainment and leisure atmospherics, the red cultural atmospherics is more conducive to improving tourists’ red tourism experience, and the improvement of red tourism experience will further help increase tourists’ confidence in Chinese culture. This study explores the impact of tourists’ perception of red cultural atmospherics on their confidence in Chinese culture from two perspectives: tourists’ perception of red education and their red cultural identity.

#### The mediating role of tourists’ perception of red education

Red tourism in China is used for developing economy, educating tourists, protecting environment and reducing poverty. The educational function of red tourism mainly reflected in patriotic education for tourists clearly distinguishes red tourism from other tourism activities. Thus, China’s General Secretary Xi Jinping repeatedly reiterated that the main goal of red tourism development in China is to promote patriotism and popularization of revolutionary history that led the CPC to achieve national liberation, independence, rejuvenation, and prosperity. Based on this, scholars define educational function of the red tourism as the ideological and political education for tourists through red tourism activities (Wall and Zhao, 2017; Liu et al., 2018), which is about improving tourists’ ideological and political quality and strengthening their party identification and national identity through the display, publicity and promotion of the red revolutionary history, revolutionary deeds and revolutionary spirit related to the red tourism destination (Zuo, 2014; Zhao and Timothy, 2015; Zuo et al., 2016; Wall and Zhao, 2017). The perception of red education means that tourists can intuitively feel and receive revolutionary and patriotic education in red tourism destinations through such activities as visiting revolutionary relics, listening to the deeds of real revolutionary heroes, watching historical photos and documentaries, and participating in ritual experience (Caraba, 2011; Liu et al., 2018; Shen et al., 2021; Tang et al., 2021).

Red cultural atmospherics is created by certain stimuli in the settings and contexts of red tourism destinations. According to the S-O-R paradigm, the stimuli from red tourism destinations will affect tourists’ internal feelings and trigger a response in them. Since there are various stimuli in a red tourism destination, one stimulus may be highly influential, whilst another may have no influence at all. Therefore, it is more appropriate to categorize the stimuli in a red tourism destination according to the information rate of the environment. Information rate refers to the spatial and temporal relationships among different stimuli in a specific setting (Jalil et al., 2016). Due to the distinctive characteristics of red tourism, the most influential stimulus in a red tourism destination is its red cultural atmospherics.

When tourists visit Wuxiang County, the red cultural atmospherics becomes their direct experience environment. This environmental stimulus will have a direct impact on their attitudes and behaviors. For instance, when tourists wear red military uniforms and eat sorghum rice, they can feel the atmosphere of the revolutionary era. Also, when interacting with local residents who reflect the Taihang spirit in their words and deeds, tourists will truly feel the grandness of the red revolution and be awed by the spirit of the heroes, which will change tourists’ attitudes and behaviors. As stated above, the core purpose of red tourism development in China is to give full play to its educational function. Tourists’ perception of red education in red tourism destinations is the core component of their red tourism experience. Previous studies have shown that the red cultural atmospherics of Jinggangshan has a significant impact on tourists’ red tourism experience (Tang et al., 2021). Therefore, this study infers that the red cultural atmospherics of a destination also has a significant positive impact on tourists’ perception of red education. Hence, this paper puts forward the following hypothesis:

*H1a-c: The red cultural atmospherics composed of physical atmospherics, spiritual atmospherics and institutional atmospherics significantly influences tourists’ perception of red education.*

Tourists’ perception of red education is formed after they acquire more knowledge about China’s revolutionary history and gain an in-depth understanding of the Taihang spirit. In this process, tourists will better recognize the history about how the CPC led Chinese people to achieve national liberation, independence, rejuvenation and prosperity. According to informal interviews with tourists in Wuxiang County, knowing the history of the founding and struggle of the Chinese nation will make tourists proud of being Chinese. In an era where national identity is becoming increasingly unstable, tourists’ pride in being Chinese will help increase their confidence in Chinese culture. A large number of qualitative studies argue that the perception of red education helps boost tourists’ confidence in Chinese culture (Li et al., 2021; Yan and Ye, 2022). Therefore, this paper postulates that:

*H2: Tourists’ perception of red education significantly influences their Chinese cultural self-confidence.*

#### The mediating role of tourists’ red cultural identity

Culture is understood as the aggregate of knowledge, values, beliefs, norms and practices shared or widely distributed in a given population (Negy et al., 2003). According to Wan and Chew (2013), cultural identity is part of an individual’s self-definition that signals the individual’s psychological connection with a certain culture. It provides a sense of common origin as well as common beliefs and values and serves as the basis of self-definition (Kim, 2007). In the traditional Chinese society, most people shared the same traditional Chinese cultural identity. However, since the implementation of the reform and opening-up policy in 1978, the popularity of western scientific education and rapid urbanization in China resulted in a multicultural environment for Chinese people. More and more people, especially the younger generation, possessed cross-cultural identity or even western cultural identity because of their insufficient understanding of the Chinese culture. Wan and Chew (2013) described the development of an individual’s cultural identity as the multi-faceted interaction tied to his or her experiences with a certain culture. Specifically, people improve their cultural identity while they acquire more knowledge about the cultural norms, beliefs, values and practices of a given cultural community and attribute such knowledge to the said cultural community.

Tourists’ red cultural identity is their psychological connection with red culture. Under the leadership of the Communist Party of China, the Chinese nation has made great achievements from gaining independence to becoming rich and strong. However, the simple passage of time has prevented many people in China from possessing such red cultural knowledge. Though people’s expression of red cultural knowledge can enhance their red cultural identity, sources of information and knowledge on red culture are relatively scarce. Red tourism destinations exist as the preservation spaces for red culture. When tourists visit red tourism destinations, their interactions with red tourism heritage sites will help them gain more knowledge and information about the red cultural values, beliefs and practices. Their psychology of seeing and attaching values and meanings to red cultural knowledge will influence their sense of belonging to the Chinese nation and their wellbeing. Such perceptions can also subconsciously impact their positive judgment on red culture, which contributes to shaping their red cultural identity. Acquisition of red cultural knowledge also provides tourists with the cultural content needed for building red cultural identity. General concerns about tourism and cultural identity have been discussed in existing literature, and the results suggest that tourism, especially cultural heritage tourism, undoubtedly helps strengthen cultural identity (Dabamona et al., 2021). In the field of red tourism research, scholars also found that red tourism helps strengthen tourists’ red cultural identity (Cui, 2018; Zhang and Ma, 2020). Based on existing literature, the red cultural atmospherics in a red tourism destination has a significant impact on tourists’ red tourism experience (Tang et al., 2021) and tourists’ perception of red education is the core of their red tourism experience. Hence, this paper proposes the following two hypotheses:

*H3a-c: The red cultural atmospherics composed of physical atmospherics, spiritual atmospherics and institutional atmospherics significantly influences tourists’ red cultural identity.*

*H4: Tourists’ perception of red education significantly influences their red cultural identity.*

As we know, cultural identity provides a sense of common origin as well as common beliefs and values and serves as the basis of self-definition. However, along with the market-oriented development of the Chinese economy in the reform-era, the country’s once highly political revolutionary arts and cultural realm has been transformed into commercialized mass culture. More and more people underestimate the value of Chinese culture they were lack of experience with the red culture atmospherics. The development of Chinese cultural identity is the process by which Chinese people establish emotional and psychological connection with Chinese cultural genes, which means they need to have a better understanding of the value of Chinese culture. Since red culture is one of the three main genes of Chinese culture, with the deepening of tourists’ red cultural identity, they have a better understanding of the founding of People’s Republic of China and the Communist Party, which will help to enhance their confidence in Chinese culture. Therefore, this paper argues that tourists’ red cultural identity will improve their Chinese cultural self-confidence. Also, a substantial amount of research argues that cultural identity is the premise of cultural confidence (Nelson, 1998; Srebrnik, 2000; Eriksen et al., 2017; Zhang, 2020; Yan and Ye, 2022). Hence, this paper postulates that:

*H5: Tourists’ red cultural identity significantly influences their Chinese cultural self-confidence.*

**FIGURE 1:**
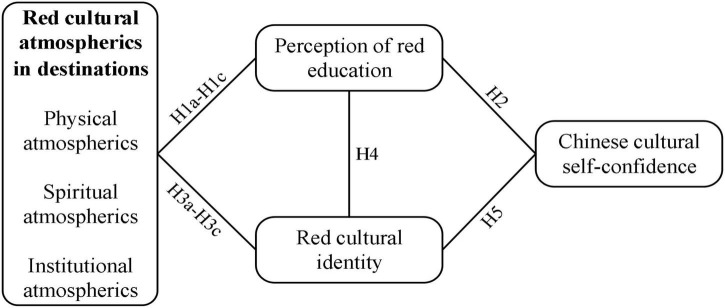
Hypothesized theoretical model.

Based on the concept of cultural atmospherics, identity theory and relevant literature, a conceptual framework is constructed below (Figure 1).

## Research methods

### Measurement items of variables

All measurement items were drawn from the literature on red tourism and cultural self-confidence. Tourists’ perception of Wuxiang’s red cultural atmospherics was measured as a multidimensional construct composed of physical atmospherics, spiritual atmospherics and institutional atmospherics, and all the items were adapted from Xu et al. (2016) and Xu and Zhu (2016). Items used to measure tourists’ perception of red education in Wuxiang County were evaluated with reference to the studies by Li et al. (2020) as well as Zhou and Tang (2022). Tourists’ red cultural identity measures were based on the research conducted by Tang et al. (2021). Chinese cultural self-confidence measures were drawn from the study carried out by Zhou and Bi (2020). Respondents were asked to describe their perceptions of the abovementioned measurement scales on a 7-point Likert scale ranging from 1 (strongly disagree) to 7 (strongly agree).

The survey questionnaire also included seven questions pertaining to tourists’ demographic characteristics (e.g., gender, age, and education level), tourists’ number of visits in Wuxiang, tourists’ motivation for red tourism, evaluation of the most impressive experience in their tours to Wuxiang and tourists’ place of residence.

### Research site

The data were collected from the holy land of Wuxiang County in Changzhi City, Shanxi province, China. Wuxiang County, as one of the birthplaces of Taihang spirit in China, covers an area of 1,610 square kilometers with a registered population of 210,000. It has inherited China’s rich red revolutionary culture and has a lot of famous red cultural resources such as the Eighth Route Army Taihang Memorial Hall, the Eighth Route Army Cultural Park, Wangjiayu Eighth Route Army Headquarters, Taihang Cadre College and Taihang Youth Military Academy. Today, Wuxiang County is a nationally renowned tourist destination for patriotic education. With its revolutionary history and rich red culture, Wuxiang was selected as one of the first “Chinese Red landmarks” by China’s Red Culture Research Association and Red Tourism Development Professional Committee in February 2017. It is also an important part of the Taihang cultural tourism brand in Shanxi Province and the core red tourism resources gathering area in the Taihang Mountains.

With the continuous development of red tourism in recent years, Wuxiang County has established a well-known red tourism brand, which has become the engine of its tourism development. However, the rapid progress of tourism in Wuxiang is attributed to not only its red cultural relics but also other natural and cultural resources. In other words, Wuxiang’s red tourism is a type of big red tourism characterized by such combinations as the combinations of red and green (red and nature-based tourism), red and folk (red and folk cultural tourism), as well as red and history (red and heritage tourism). As a result, the red cultural atmospherics is not so strong, resulting in ineffective patriotic education and tourists’ lack of Chinese cultural self-confidence. Therefore, taking Wuxiang County as the research site may be conducive to the inheritance of Wuxiang’s red culture gene and offering managerial implications to scientifically improve Wuxiang’s red cultural atmospherics to strengthen tourists’ confidence in Chinese culture.

### Sampling and data collection

To improve the questionnaire’s construct and content validity, a pilot survey was conducted. The researchers first invited some college students who had visited Wuxiang County in the past half year to read the measurement items to make sure there were no linguistic or cognitive ambiguities. After that, a preliminary survey was conducted on researchers’ friends and relatives who had recently traveled to Wuxiang County. A total of 96 valid questionnaires out of the 111 distributed questionnaires were recovered. The reliability and validity of the recovered questionnaires were tested. The results showed that the Cronbach’s α of each latent variable was greater than 0.7, and the factor loading of each measurement item was greater than 0.7 at the significance level of 0.01, indicating that the scales had good reliability and validity and could be used for large-scale formal surveys.

The official survey of this study was conducted among tourists from May 2022 to June 2022 in Wuxiang County. One researcher and one graduate student majoring in Tourism Management came to Wuxiang to invite the tourists who had completed their Wuxiang tour to fill in the questionnaires at the exits of the red tourist attractions as well as the parking lots in Wuxiang. A total of 328 valid questionnaires were recovered and were used for empirical analysis. Among the valid questionnaires, men accounted for 45.7%, and females 54.3%. A total of 59.1% of the respondents were aged between 26 and 40 years old, and the majority of the respondents were highly educated (68.3% had obtained a bachelor’s degree and 16.8% had obtained a master’s degree). The data revealed that 46% of the respondents visited Wuxiang for the first time, and 25.6% for the second time (Table 1). More than 50% of the tourists came to visit Wuxiang to cherish the memory of the revolutionary heroes, experience the revolutionary cultural atmosphere, and learn about the revolutionary history. The most impressive part of the trip to Wuxiang was hearing the stories of revolutionary heroes and seeing the red historical and cultural relics there.

**TABLE 1:** Demographic characteristics of the sample.

Variable	*n*	%	Variable	*n*	%
**Gender**			**Number of visits**		
Male	150	45.7	One	151	46
Female	178	54.3	Two	84	25.6
**Age (years old)**			Three	42	12.8
Less than 25	72	22	More than three	51	15.5
25-40	194	59.1	**Education Level**		
41-50	54	16.5	Less than High School	7	2.1
51 or older	8	2.4	High school/Technical secondary school	42	12.8
**Originating Area**			Bachelor’s degree	224	68.3
Wuxiang County	37	11.3	Master’s degree	55	16.8
Changzhi city	83	25.3			
Shanxi province	187	57			
Other provinces	21	6.4			

## Results

According to Hair et al. (2012), partial least squares structural equation modeling (PLS-SEM) is perfectly suitable for predictive research and theoretical development, which is similar to that of this study. Additionally, strictly normal distribution of measured variable data is not required in PLS-SEM (Hwang et al., 2010). Therefore, PLS-SEM was used to test the reliability, validity, and causality of the theoretical model in this study.

Following the procedure of the structural equation modeling (SEM), a two-step approach was used to analyze the data. Specifically, the overall measurement model was tested first, followed by the assessment of the structural model.

### Measurement model

Partial least squares structural equation modeling algorithm in Smart PLS 3.0 was used to evaluate the overall measurement model’s reliability and validity, since there are no distributional requirements of the measured variables’ data in PLS-SEM. The results are presented in Table 2 below. Reliability analysis reflects a scale’s internal consistency and reliability. A model is considered reliable when the Cronbach’s α and composite reliability of each latent construct are equal to or greater than 0.70 (Martin Wetzels, 2009; Fornell and Larcker, 2012; Lv et al., 2020). In this study, Cronbach’s α and composite reliability range from 0.959 to 0.982, exceeding the threshold value of 0.7, indicating that the reliability of the measurement model is satisfactory. Convergent validity was examined via the tests of factor loadings of the observed items and average variance extracted (AVE) of the latent constructs. The factor loadings of the observed items for each latent construct in this study range from 0.880 to 0.968, all exceeding the threshold of 0.7. Additionally, the AVE value of each latent construct ranges from 0.834 to 0.918, larger than the acceptable requirement of 0.5, indicating that the scale exhibited sufficient reliability and convergent validity.

**TABLE 2:** Reliability and validity of the measures.

Latent variables and items	Descriptive statistics		Convergent validity		Reliability	
	Mean	*SD*	Factor loading	AVE	CR	Cronbach’s α
**(1) Physical atmospherics (PA)**	4.670	1.445		0.834	0.972	0.967
The architecture in Wuxiang can demonstrate the characteristics of Taihang spirit.			0.909***			
The red tourism landscapes in Wuxiang can demonstrate the characteristics of Taihang spirit.			0.931***			
I can often hear red-themed music usually in Wuxiang.			0.936***			
Wuxiang can provide Taihang cave and other local B and B service.			0.918***			
The catering snacks in Wuxiang can reflect its revolutionary history.			0.894***			
Tourist souvenirs in Wuxiang embody the Taihang spirit.			0.922***			
The main traffic routes into Wuxiang can show the Taihang spirit.			0.880***			
**(2) Spiritual atmospherics (SA)**	4.869	1.507		0.880	0.981	0.977
Wuxiang can truly show the Taihang revolutionary spirit.			0.942***			
The residents in Wuxiang are hospitable.			0.951***			
The residents in Wuxiang have a strong sense of pride in Taihang spirit.			0.932***			
The residents in Wuxiang will actively promote Taihang spirit.			0.918***			
The people in the red scenic spots show a positive mental state.			0.942***			
The people in the red scenic spots will actively promote Taihang spirit.			0.936***			
Wuxiang represents China’s revolutionary history authentically.			0.947***			
**(3) Institutional atmospherics (IA)**	4.638	1.555		0.900	0.982	0.978
Tourism operation in Wuxiang is orderly.			0.941***			
The people in the red scenic spots in Wuxiang act in good manners.			0.953***			
The people in the red scenic spots in Wuxiang provide excellent service.			0.945***			
The public order in Wuxiang is excellent.			0.952***			
The environment in Wuxiang is harmonious.			0.952***			
The environment in Wuxiang is clean and tidy.			0.949***			
**(4) Perception of red education (PRE)**	5.059	1.427		0.899	0.973	0.963
Traveling to Wuxiang offers a red education opportunity to me.			0.949***			
Traveling to Wuxiang strengths my belief in communism.			0.953***			
Traveling to Wuxiang has enriched my knowledge of China’s revolutionary history.			0.952***			
Traveling to Wuxiang has deepened my understanding of the Taihang spirit.			0.938***			
**(5) Red cultural identity (RCI)**	5.253	1.448		0.918	0.978	0.970
I have strengthened Chinese red cultural identity during this tour.			0.958***			
I have strengthened national identity during this tour.			0.968***			
I have a strong sense of pride in our Chinese nation during this tour.			0.953***			
I have strengthened the Communist Party identity during this tour.			0.953***			
**(6) Chinese cultural self-confidence (CSC)**	5.509	1.337		0.891	0.970	0.959
Traveling to Wuxiang has strengthened my confidence in Chinese culture.			0.947***			
I firmly believe that China can cope with and address any difficulties we encounter after visiting Wuxiang.			0.955***			
I am full of pride in Chinese culture.			0.937***			
I firmly believe that Chinese culture is unique compared with other cultures after visiting Wuxiang.			0.936***			

***p < 0.001.

The Fornell-Larcker criterion and the examination of cross-loadings are the dominant approaches for evaluating discriminant validity in PLS-SEM (Urbach and Ahlemann, 2010). However, Henseler et al. (2015) pointed out that these two approaches do not reliably detect the discriminant validity in common research situations. Therefore, they proposed the heterotrait-monotrait ratio of correlations (HTMT) as a new approach to assess discriminant validity together with the two dominant approaches. In our research, each item’s factor loading on its variable is greater than all its loadings on other variables. The square root of AVE value of each latent variable is larger than the correlation coefficients with other latent variables (Table 3), suggesting that the discriminant validity of the constructs can satisfy the first two requirements. According to Table 4, the HTMT ratio is less than the threshold of 0.85. All these indicated that the scales in the present study have good discriminant validity.

**TABLE 3:** The Fornell-Larcker discriminant validity of constructs.

Constructs	1	2	3	4	5	6
(1) Physical atmospherics	[0.913]					
(2) Spiritual atmospherics	0.691	[0.938]				
(3) Institutional atmospherics	0.798	0.731	[0.949]			
(4) Perception of red education	0.764	0.744	0.806	[0.948]		
(5) Red cultural identity	0.763	0.759	0.790	0.808	[0.958]	
(6) Chinese cultural self-confidence	0.708	0.667	0.666	0.745	0.809	[0.944]

[] represents the square root of AVE of each latent construct.

**TABLE 4:** Heterotrait-monotrait ratio of correlations (HTMT) discriminant validity of constructs.

Constructs	1	2	3	4	5	6
(1) Physical atmospherics	–					
(2) Spiritual atmospherics	0.708					
(3) Institutional atmospherics	0.820	0.747				
(4) Perception of red education	0.791	0.766	0.830			
(5) Red cultural identity	0.787	0.778	0.810	0.836		
(6) Chinese cultural self-confidence	0.735	0.688	0.687	0.775	0.839	–

### Structural model

After the measurement model was successfully validated, the structural model was analyzed by Smart PLS3.0. The endogenous latent variables’ coefficients of determination (*R*^2^) measure the relationship of the latent variables’ explained variance to their total variance (Henseler, 2010; Urbach and Ahlemann, 2010; Lv et al., 2022; Yang et al., 2022). According to Chin (1998), the explanatory power of a structural model is substantial when the value of *R*^2^ is approximately 0.670, average when the value of *R*^2^ is around 0.333, and weak when the value of *R*^2^ is 0.190. In our model, the value of *R*^2^ ranges from 0.679 to 0.747 (Table 5), exceeding the substantial threshold of 0.670, indicating a high level of explanatory power of our research.

**TABLE 5:** Structural model results and effects sizes (*f*^2^).

Endogenous variable	Predictor variable	*R* ^2^	Path coefficient	*t* value	*P-value*	*f* ^2^
Perception of red education	Physical atmospherics	0.722	0.253	4.816	0.000	0.078
	Spiritual atmospherics		0.274	5.866	0.000	0.117
	Institutional atmospherics		0.403	6.707	0.000	0.176
Red cultural identity	Physical atmospherics	0.747	0.189	2.790	0.005	0.044
	Spiritual atmospherics		0.240	4.415	0.000	0.088
	Institutional atmospherics		0.206	2.451	0.014	0.043
	Perception of red education		0.320	3.837	0.000	0.112
Chinese cultural self-confidence	Perception of red education	0.679	0.264	3.997	0.000	0.075
	Red culture identity		0.596	9.123	0.000	0.384

**FIGURE 2:**
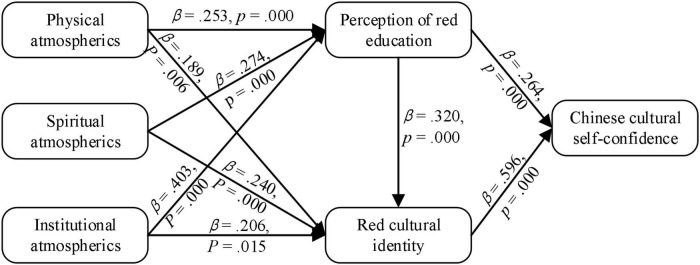
The path analysis results of structural equation modeling (SEM).

Path coefficients can measure the strength of the relationship between latent variables of the model, where the significant value should be at least 0.05 (Urbach and Ahlemann, 2010). The path coefficients and their significant values are listed in Figure 2. It can be seen that the red cultural atmospherics of Wuxiang significantly affects tourists’ perception of red education (β*_1*a*_* = 0.253, *p* = 0.000; β*_1*b*_* = 0.274, *p* = 0.000; β*_1*c*_* = 0.403, *p* = 0.000) and red cultural identity (β*_3*a*_* = 0.189, *p* = 0.006; β*_3*b*_* = 0.240, *p* = 0.000; β*_3*c*_* = 0.206, *p* = 0.015). Hypotheses 1 and 2 are supported. Tourists’ perception of red education significantly affects their red cultural identity (β*_2_* = 0.320, *p* = 0.000), hypotheses 3 is supported. Tourists’ perception of red education (β*_4_* = 0.264, *p* = 0.000) and red cultural identity (β*_5_* = 0.596, *p* = 0.000) significantly affect their Chinese cultural self-confidence. Hypotheses 4 and 5 are supported.

The effect size of each path which measures if an independent latent variable has a substantial impact on a dependent latent variable in the structural equation model is evaluated by means of Cohen’s *f* ^2^ (Cohen, 1988). Values for *f*^2^ in our research range from 0.043 to 0.384, greater than the minimum threshold of 0.02 (Cohen, 1988; Chin, 1998), which means the predictor variables have different levels of effect on the endogenous latent variables.

The structural model’s predictive relevance was assessed by *Q*^2^. The *Q*^2^ was assessed to test the predictive capacity of the model. Both the endogenous constructs’ *Q*^2^ values (0.642 for perception of red education, 0.680 for red cultural identity, and 0.599 for Chinese cultural self-confidence) are larger than 0 (Stone, 1974; Chin, 1998), which means the predictive relevance of the model is significant. At last, the overall goodness-of-fit (GoF) of the model in this study was calculated as 0.79, higher than the judgment standard of strong goodness-of-fit (GoF_*large*_ > 0.36) (Martin Wetzels, 2009), indicating that the model in this study is satisfactory.

### The mediating effects of tourists’ perception of red education and their red cultural identity

Table 6 shows the results of a mediation analysis with tourists’ perception of red education and their red cultural identity as parallel mediators (Hayes, 2017, model 4: 5,000 bootstrapped samples; IV = red cultural atmospherics, DV = Chinese cultural self-confidence, M1 = tourists’ perception of red education, M2 = tourists’ red cultural identity). They indicate that the direct effect of red cultural atmospherics on tourists’ Chinese cultural self-confidence is not significant (95% CI [–0.0449,0.2226]), which means that the stimuli from the cultural atmospherics of red tourism destinations can not directly lead to the development of tourists’ Chinese cultural self-confidence. The indirect effect of both tourists’ perception of red education (index of mediation = 0.1825, 95% CI [0.0661,0.3367]) and their red cultural identity (index of mediation = 0.4602, 95% CI [0.3301,0.5850]) are positive and significant.

**TABLE 6:** Results of the mediating effect test.

Dependent variable	Effect path	Effect	BootSE	LLCI	ULCI	*P-value*
Chinese cultural self-confidence	Direct effect	0.0888	0.0680	–0.0449	0.2226	0.1921
	Total indirect effect	0.6426	0.0791	0.4981	0.8096	0.000
	Indirect effect of tourists’ perception of red education	0.1825	0.0690	0.0661	0.3367	0.000
	Indirect effect of tourists’ red cultural identity	0.4602	0.0649	0.3301	0.5850	0.000

## Discussion and conclusion

### Discussion

As predicted, findings confirm the existence of a significant positive relationship between red cultural atmospherics and tourists’ Chinese cultural self-confidence. This conclusion is consistent with other scholars’ research conclusion that the atmospherics in a restaurant or store or festival had a significant positive effect on customers’ positive attitude and behavioral intentions (Baker et al., 2002; Heung and Gu, 2012; Uhrich and Benkenstein, 2012; Vilnai-Yavetz et al., 2021). This study contributes to the atmospherics literature in marketing. Bitner (1992) stated that in marketing area there was a surprising lack of empirical research or theoretically based frameworks addressing the role of physical surroundings in consumption settings, which resulted in managers lack of special knowledge in planning, building, and changing an organization’s physical surroundings in an attempt to control its influence on patrons. Cultural atmospherics is the specific design in culture in the overall atmospherics. Our findings show that destinations’ red cultural atmospherics can generate positive tourist experience, which highlights that in the era of the integration of culture and tourism, all destinations, especially cultural heritage sites and other rural tourism destinations, must attach greater importance to the design of their cultural atmospherics for tourists.

This study also contributes to the red tourism literature by providing empirical support for the development of tourists’ confidence in Chinese culture through red tourism in China. Most of the existing studies on red tourism only qualitatively analyze the relationship between red tourism and Chinese cultural self-confidence (Wall and Zhao, 2017; Zhang and Ma, 2020; Tang et al., 2021). This study provides empirical support for the relationship between destinations’ red cultural atmospherics and tourists’ confidence in Chinese culture, which urges the government to prioritize the development of red tourism in tourism planning.

In addition, this study offers managerial implications for red tourism destinations’ planning and scientific improvement of the red cultural atmospherics based on the empirical results. Particularly, the findings clearly indicate that the red cultural atmospherics in Wuxiang is not intense. The means of physical atmospherics, spiritual atmospherics and institutional atmospherics are respectively 4.67, 4.869, and 4.638 points, suggesting that the red cultural atmospherics in Wuxiang is in urgent need of scientific improvement to increase tourists’ confidence in Chinese culture through red tourism.

### Practical implications

Research findings reveal that tourists’ perceptions of Wuxiang’s red cultural atmospherics can boost their confidence in Chinese culture, which provides several implications for red tourism destination managers to better promote tourists’ Chinese cultural self-confidence through red tourism.

Red tourism destinations’ planners and managers should attach equal importance to the making of their red cultural atmospherics as well as scenic spots design in the process of planning and development. Any tourism destination can provide tourists with various tourism activities to participate in. Thus, in order to meet the diverse interests of tourists, many red tourism destinations make a whole atmosphere of entertainment and vacation in the red tourism planning and development, which is very detrimental to the red tourism experience of tourists. This study suggests that destinations must pay attention to the making of the overall red cultural atmospherics to enhance tourists’ confidence in Chinese culture. For example, red tourism destinations can create coherent physical atmospherics, spiritual atmospherics and institutional atmospherics across the destination by implementing various plans such as designing the physical environment with red-themed culture, training the local residents and tourism workers to truly showcase the red revolutionary spirit, and designing various authentic, red-themed activities. Wuxiang County, as the birthplace of Taihang revolutionary spirit, should highlight the Taihang spirit in the overall physical environment and nurture local residents and tourism practitioners with Taihang spirit. Through the improvement of the red cultural atmospherics, red tourism destinations can present an authentic red cultural environment for tourists to experience. One issue worth noting here is the potential excessive entertainment of red tourism destinations, which could sacrifice the authentic red cultural atmospherics. Besides, the differences in the effects of physical atmospherics, spiritual atmospherics and institutional atmospherics on tourists’ Chinese cultural self-confidence provide an opportunity for red tourism destinations to give priority to improving the institutional atmospherics.

Meanwhile, our research found that red cultural atmospherics can not directly lead to the development of tourists’ Chinese cultural self-confidence, which enlightens red tourism destinations should not only pay attention to the making of red cultural atmospherics, but also pay attention to tourists’ red tourism experience, so as to achieve the purpose of the development of tourists’ confidence in Chinese culture through red tourism. Empirical results uncovered significant mediating effects of tourists’ perception of red education and their red cultural identity, which draw the attention of red tourism destinations’ managers to the improvement of tourists’ perception of red education and their red cultural identity. Red tourism is a special culture-themed tourism activity in China. The informal survey in Wuxiang County revealed that the quality of tour guides’ interpretation is an important factor affecting tourists’ perception of red education and red cultural identity. For example, tourists said they can feel the spirit of revolutionary heroes spontaneously when tour guides explained the stories of general Zuo Quan and commander Zhu De. However, the contents delivered by and methods of delivery of some tour guides in some red scenic spots are in urgent need of improvement. Therefore, red tourism destinations must pay attention to the training of the tour guides and other service personnel to effectively provide tourists with red revolutionary knowledge and culture to improve the quality of their service. In addition, tourists said that their participation and immersion in red-themed activities can enliven their patriotism education perception and their red cultural identity, which enlighten Wuxiang to design various red-themed activities for tourists.

### Conclusion

In the era of economic and cultural globalization, Chinese cultural self-confidence is an important factor affecting the prosperity and development of China. This paper established a model to explore the development of tourists’ confidence in Chinese culture through red tourism, especially the effect of the red cultural atmospherics in a red tourism destination. PLS-SEM was used to empirically test the impact, and the mediating roles of perception of red education and red cultural identity were also validated. The main findings are as follows:

(1) The red cultural atmospherics in a red tourism destination positively affects tourists’ perception of red education and their red cultural identity. As mentioned above, red cultural atmospherics is composed of physical atmospherics, spiritual atmospherics and institutional atmospherics (Xu et al., 2016). All the three dimensions can positively affect tourists’ perception of red education and their red cultural identity. However, the three dimensions exert different influences. In terms of tourists’ perception of red education, the most influencing factor is the institutional atmospherics (β = 0.403, *p* = 0.000), followed by spiritual atmospherics (β = 0.274, *p* = 0.000) and physical atmospherics (β = 0.253, *p* = 0.000). The order of influence of these three components on tourists’ red cultural identity from high to low is spiritual atmospherics (β = 0.240, *p* = 0.000), institutional atmospherics (β = 0.206, *p* = 0.015) and physical atmospherics (β = 0.189, *p* = 0.006).
(2) Tourists’ perception of red education positively affects tourists’ red cultural identity and their Chinese cultural self-confidence by mediating the effects of red cultural atmospherics in a red tourism destination on tourists’ red cultural identity and their Chinese cultural self-confidence. Since the educational function is the purpose for the Chinese government to develop red tourism (Xu, 2015; Zuo et al., 2016; Wall and Zhao, 2017); tourists’ perception of red education constitutes the core component of tourists’ red tourism experience. As an important stimulus in red tourism destination’s settings and contexts, the red cultural atmospherics of the red tourism destinations affects tourists’ red cultural identity and Chinese cultural self-confidence through tourists’ perception of red education.
(3) Tourists’ red cultural identity positively affects their Chinese cultural self-confidence. A substantial amount of qualitative literature suggests that cultural identity is the premise of cultural confidence (Cui, 2018; Wang et al., 2018; Yan and Ye, 2022). This empirical study supports this statement. As an important part of Chinese culture, the improvement of tourists’ identification with red culture helps build their confidence in Chinese culture (Elmashhara and Soares, 2022).

### Limitations and suggestions for future research

To help tourists understand Chinese culture and build their Chinese cultural self-confidence relying on red tourism is an important task for the development of red tourism during the 14th Five-Year Plan (2021–2025) period. In this context, this paper explored and empirically tested the mechanism by which red cultural atmospherics in a red tourism destination impacts tourists’ Chinese cultural self-confidence. However, this study also has the following limitations.

As the historic and cultural relics of the Chinese Communist Party, red tourism destinations are accredited as an essential means to carry out patriotic education and build tourists’ Chinese cultural self-confidence. Given the special characteristics of red tourism destinations, this paper focused on the impact of the overall red cultural atmospherics of the red tourism destinations on tourists’ Chinese cultural self-confidence. The effects of additional characteristics of red tourism destinations, such as the landscape design of red tourism sites and the interpretation of red culture in red scenic spots, still require more extensive discussions in future research.

This study tested the partial mediating roles of tourists’ perception of red education and red cultural identity in the effect of red cultural atmospherics on tourists’ Chinese cultural self-confidence. There is a strong need to understand other psychological processes that underpin tourists’ Chinese cultural self-confidence. Whether there are other mediating mechanisms remains to be further explored by future research.

We know red tourism development processes vary significantly from place to place. This study is restricted to Wuxiang County, Shanxi Province in China only, and thus the results may not be applicable in other places. Future studies are needed to test the applicability of the model proposed in this study to other red tourism destinations in China.

Finally, Chinese cultural self-confidence is an important factor affecting the prosperity and development of China in the era of economic and cultural globalization. Ministry of Culture and Tourism of the People’s Republic of China hopes to turn tourism into a process for tourists to understand Chinese culture and build their Chinese cultural self-confidence in the 14th Five-Year Plan period. In this context, cultural atmospherics making is an important strategy for destinations’ tourism planning and development, especially for rural tourism destinations and cultural heritage sites. Therefore, how to scientifically make the cultural atmospherics of different destinations remains to be studied in future.

## Data availability statement

The raw data supporting the conclusions of this article will be made available by the authors, without undue reservation.

## Author contributions

XZ: propose research hypotheses, build models, and write the first draft. YG: collect and analyse data. XX: collect literatures and put forward policy suggestions. CL: review and editing. FZ: contribute to the theoretical building of the manuscript and take responsibility for manuscript submission. All authors contributed to the article and approved the submitted version.
